# Supplementary material for: Usability and feasibility of a digital cognitive screening tool measuring older adults’ early postoperative neurocognitive recovery: a protocol for a pilot study
Source: BMJ Open. 2023 Jul 21;13(7):e070404. doi: 10.1136/bmjopen-2022-070404 (PMC10364180; doi:10.1136/bmjopen-2022-070404)
Supplement: Author's manuscript [file bmjopen-2022-070404.draft_revisions.pdf]

BMJ Open is committed to open peer review. As part of this commitment we make the peer review history of every article we publish publicly available.

When an article is published we post the peer reviewers' comments and the authors' responses online. We also post the versions of the paper that were used during peer review. These are the versions that the peer review comments apply to.

The versions of the paper that follow are the versions that were submitted during the peer review process. They are not the versions of record or the final published versions. They should not be cited or distributed as the published version of this manuscript.

BMJ Open is an open access journal and the full, final, typeset and author-corrected version of record of the manuscript is available on our site with no access controls, subscription charges or pay-per-view fees (http://bmjopen.bmj.com).

If you have any questions on BMJ Open's open peer review process please email info.bmjopen@bmj.com

BMJ Open

Usability and feasibility of a digital cognitive screening tool measuring older adults' early postoperative neurocognitive recovery: A protocol for a pilot study

Journal:	*BMJ Open*
Manuscript ID	bmjopen-2022-070404
Article Type:	Protocol
Date Submitted by the Author:	21-Nov-2022

Complete List of Authors:	Amirpour, Anahita; Karolinska Institutet, Neurobiology, Care Sciences and Society<br>Eckerblad, Jeanette; Karolinska Institute,<br>Thorell , A; Karolinska institutet Department of Clinical Sciences Intervention and Technology,<br>Bergman, Lina; Karolinska Institute,<br>Nilsson, Ulrica; Karolinska Institutet, Department of Neurobiology, Care Sciences and Society, Karolinska Institutet Perioperative Medicine and Intensive Care; Karolinska Universitetssjukhuset, Perioperative Medicine and Intensive Care

Keywords:	Adult anaesthesia < ANAESTHETICS, GERIATRIC MEDICINE, Adult surgery < SURGERY

SCHOLARONE™
Manuscripts

Usability and feasibility of a digital cognitive screening tool measuring older adults' early postoperative neurocognitive recovery: A protocol for a pilot study.

Anahita Amirpour[1], Jeanette Eckerblad [1], Anders Thorell [2], Lina Bergman [1], Ulrica Nilsson [1,3].

1. Department of Neurobiology, Care sciences and Society. Karolinska Institutet, Stockholm, Sweden.
2. Department of Clinical Science at Danderyds hospital, Karolinska Institutet, and Department of Surgery, Ersta Hospital, Stockholm, Sweden
3. Perioperative medicine and intensive care, Karolinska University Hospital, Stockholm, Sweden.

Trial registration number NCT05564195

Issue date 220801

Protocol amendment: 10

Authors: A.A, JE, AT, LB, UN

Revision chronology:

Corresponding Author

Anahita Amirpour

Division of Nursing

Department of Neurobiology, Care sciences and Society

Karolinska Institutet

Alfred Nobels allé, C4

14183 Huddinge, Sweden

Email: anahita.amirpour@ki.se

Phone: +46 8524 888 73

For peer review only - http://bmjopen.bmj.com/site/about/guidelines.xhtml

Key words: postoperative neurocognitive recovery, postoperative cognitive decline, geriatric medicine, adult anaesthesia.

Word count: 2979

For peer review only

For peer review only - http://bmjopen.bmj.com/site/about/guidelines.xhtml

ABSTRACT

Introduction Delayed neurocognitive recovery, also identified as early postoperative cognitive decline (POCD), is a common complication after surgery, with advanced age to be the most important risk factor. As the geriatric population is increasing worldwide, and number of older adults undergoing surgery continues to rise, so will the incidence of POCD. Only a small proportion using digital cognitive tests for measuring postoperative neurocognitive performance compared to analogue tests. This study aims to evaluate a digital cognitive screening tool, Mindmore-P, in a perioperative setting by determine its feasibility and usability and to compare preoperative cognition with early postoperative neurocognitive performance. Further, to determine associations between neurocognitive performance and perioperative factors as well as to explore patients' experiences of early neurocognitive recovery.

Methods and analysis: We will include fifty patients (age ≥60 years) undergoing elective abdominal surgery under general anesthesia. Cognitive functions will be measured with Mindmore-P preoperatively and on postoperative day (POD) 1 or 2 as well as 2-3 weeks after surgery. Preoperatively, frailty, (Clinical Frailty Scale), depression (Geriatric Depression Scale-15), functional status (WHODAS) and pre-recovery status (Swedish Quality of Recovery Scale, SwQoR) will be measured. Delirium will be assessed by Nu-DESC twice a day, with start on POD 1 and until the patient is discharged from the hospital. Outcomes at 2-3 weeks postoperatively are postoperative recovery (SwQoR), depression, functional status and usability (System Usability Scale) of Mindmore-P. Postoperative recovery will also be measured POD 1 or 2. We will also explore feasibility and experience of early postoperative neurocognitive recovery with interviews approximately one month after surgery.

Ethics and dissemination This study is approved by the Swedish Ethical Review Authority (REC Reference: 2022-03593-01) and will follow the principles outlined in the 1964 Helsinki Declaration and its later amendments. Results from this study will be disseminated in peer-reviewed journals, scientific conferences and in social media.

For peer review only

Strengths and limitations of this study:

- This study follows the Medical Research Councils framework for designing and evaluating complex interventions
- This study will provide evidence for the feasibility and usability of a digital neurocognitive tool to be used in a population of older persons undergoing surgery
- This pilot study will create a knowledge base for future interventions as previous studies are lacking
- A limitation is that the study only includes two follow ups

For peer review only

For peer review only - http://bmjopen.bmj.com/site/about/guidelines.xhtml

Introduction

The geriatric population is increasing worldwide[1]. Every fifth person in Sweden today is ≥65 years, and the number of persons of 80 years and over, is estimated to increase by 50% until 2050 [2, 3]. National statistics show that approximately 700 000 people undergo in-patient surgery, and almost 50% of those are performed on people ≥ 60 years[4]. However, hospital care after surgery remains short for the afflicted population; hence a major part of the postoperative recovery occurs after discharge from the hospital which poses several risks for older people[2].

Cognitive decline in older individuals has become an area of great concern, particularly given the impact of cognitive impairment on functional capacity[5]. Cognitive functions in late adulthood decline at different pace, and some cognitive domains are more prone to faster decline, such as executive functions, speed processing and memory [6]. Delayed neurocognitive recovery, earlier identified as postoperative cognitive decline (POCD), is an early postoperative cognitive complication, included in perioperative neurocognitive disorders. Delayed neurocognitive recovery is diagnosed up to 30 days after anaesthesia and surgery [1, 7] and is characterized by a gradual decreased performance in several cognitive domains such as memory, speed processing, executive function, and attention[1, 8]. The term POCD will be used in this article when referring to earlier research.

Preoperative risk factors for developing POCD after surgery are advanced age, pre-existing cognitive decline, visual and audial impairment, ongoing infection or serious illness [9]. Other risk factors are impaired functional status, lower level of educational attainment [7, 10, 11], alcohol abuse, electrolyte disturbances either medication-induced or due to dehydration, psychoactive medications, depression and frailty [10, 11]. Although no causality has been demonstrated, POCD is associated with poor postoperative outcomes including prolonged length of hospital stay, increased morbidity / mortality, and reduced quality of life, resulting in a significant burden on the individual, their family, as well as for the healthcare system. To date there is no existing pharmacological or non-pharmacological intervention to treat postoperative neurocognitive decline or decrease the associated symptoms [7, 11]. Moreover, results have been inconclusive regarding the role of intraoperative factors such as type of anesthesia, hypotension, cerebral saturation or choice of anesthetic drugs for the development of POCD [12]. One hypothetical mechanism is that older adults' have an unregulated acute inflammatory response to surgery[13]. So far, an explicit connection between POCD and

For peer review only - http://bmjopen.bmj.com/site/about/guidelines.xhtml

neuroinflammation due to the surgical trauma has been confirmed [7, 14].The American Geriatrics society's guidelines [9] recommend that every geriatric perioperative patient with risk factors, should be assessed for their cognitive performance prior surgery. Despite the potential benefits, it seems challenging to implement such a routine in regular clinical practice. There is therefore a need to develop and implement feasible measures to assess cognitive function in a busy clinical environment [11].

The methods used for measuring postoperative neurocognitive performance vary widely between studies, with only a small proportion, 13.5%, using digital cognitive tests or a combination of analog and digital tests [15]. To cover this gap we conducted a comparability study [16] between the digital cognitive test battery Mindmore Postoperative version, (Mindmore-P), and the analog International Study Group of Postoperative Cognitive Dysfunction (ISPOCD) test battery[17]. The study included 50 cognitively healthy participants aged 60 years and older. We found that the included tests in ISPOCD and Mindmore-P were comparable. Further, the participants were overall positive to digital testing and thought that neurocognitive testing was useful and important to assess if they would undergo surgery (Manuscript in progress). The next step, following Medical Research Councils framework for designing and evaluating complex interventions[18] is to assess predefined progression criteria to reduce uncertainty around recruitment, data collection, outcomes, sample size, and analysis in a future intervention study.

Aim

The aim of this study is to pilot test Mindmore-P, in a clinical perioperative setting. To achieve this, we intend:

I) To test the recruitment process and measure attrition rate.

II) To determine the usability and feasibility of Mindmore-P.

III) To explore the trajectory of delayed neurocognitive recovery by measuring the patient's preoperative and postoperative neurocognitive performance.

IV) To investigate if there are any associations between the dependent variable; delayed neurocognitive recovery and-the independent variables; delirium, preoperative frailty, symptoms of depression, postoperative delirium, physical function, and early postoperative recovery.

V) To determine patients' experiences of early neurocognitive recovery.

For peer review only - http://bmjopen.bmj.com/site/about/guidelines.xhtml

METHODS AND ANALYSIS

Study design

This is an observational feasibility study.

Participants, recruitment, and setting

We will include 50 patients undergoing elective abdominal surgery under general anesthesia at Ersta hospital in Stockholm, Sweden. Patient recruitment is planned to start in December 2022 and end in July 2023. The sample size is guided by a reported incidence of early cognitive decline at 1–2 weeks postoperatively of approximately 13-25% [17]. At the patient's preoperative outpatient visit, a research nurse will provide oral and written information about the study. The details of the study and its potential benefits and risks will be explained carefully to the patient. If the patients agree to participate in the study, they will undergo a Mini-mental state examination (MMSE) screening with the research nurse to examine if they are eligible for the study.

Inclusion criteria:

1) ≥60 years of age undergoing elective non-cardiac surgery with general anesthesia.
2) Duration of surgery ≥60 minutes
3) MMSE score ≥24.

Exclusion criteria:

1) MMSE score of ≤23 indicating cognitive impairment [19].
2) Inability to read and speak Swedish.
3) Uncorrected severe visual or auditory disorder, disease of the central nervous system, psychiatric diseases including alcohol abuse or drug dependence, current motor impairment in dominant hand, and colour vision deficiency.

Outcomes

Cognitive tests

The intervention is the digital cognitive test battery Mindmore-P (Table 1), which will assess neurocognitive performance. The test battery measures following cognitive domains; verbal episodic memory, executive functions, visuospatial function and attention.

For peer review only - http://bmjopen.bmj.com/site/about/guidelines.xhtml

Table 1. Included tests and cognitive domains in the Mindmore-P

**Cognitive Domain**	Mindmore-P
Verbal episodic memory	Consortium to Establish a Registry for Alzheimer´s Disease (CERAD)<br><br>A 10-word verbal learning test with 3 learning trials, a recall trial after ~7 min. Words are read and visually presented on the screen and the patient replies in speech.
Executive function, visuospatial function	Trail Making Test (TMT- A & B)<br><br>Each part consists of 25 circles with letters or numbers on the screen. In part A, the circles are numbered from 1 to 25. The patient is instructed to draw a line between them in ascending order of numbers, from 1 to 25, as rapidly as possible. Part B consists of 13 digits and 12 letters to be paired, and the test subject is instructed to draw a line between them in ascending number and letter order.
Executive function, selective attention	Stroop Colour-Word Test (SCW).<br><br>24 words spelling out the name of a colour which is printed in contrasting ink colours (e.g., the word “green” printed with red ink), and the participant is asked to tell the printed colour of the word rather than the actual meaning of the word.
Executive function, visuospatial function	Symbol Digits Processing Test (SDPT).<br><br>The test consists of nine symbol–digit pairings at the top of the screen, one symbol in the middle of the screen, and a 3 × 3 number grid at the bottom of the screen. The participant is required to associate the symbol in the middle to one of the digits on the grid below. As soon as a choice is made a new symbol appears. The final score is the number of correct matches in 90 seconds

The Mindmore-P is self-administered on a touch screen tablet (10.1″ 20-30 min Windows). and is a web-application running on a full-screen Chrome browser. The test is administered by using a capacitive touchscreen with features that record detailed information such as timing, pauses, and lifting of finger from the touchscreen. Speech recognition is used in the screening of verbal memory and language.

Usability

The usability (Table 2) of Mindmore-P will be assessed with the System Usability Scale (SUS). SUS consists of 10 items to be answered on a 5-point scale from “strongly agree” to “strongly disagree”. [20]

For peer review only - http://bmjopen.bmj.com/site/about/guidelines.xhtml

Depression screening

The Geriatric Depression Scale (GDS) is a widely used instrument to screen for depression in the older adult population[21]. The GDS-15 consists of 15 items with binary answer (yes/no) to questions such as "*Do you feel helpless*?" or "*Are you in good spirits most of the time*? [22]. The cut-off score is standardized with a score of 6 to the Swedish older adult population[23]. Every participant will answer the Swedish version of the GDS-15 before surgery and on postoperative day 14 at the revisit at the hospital.

Preoperative frailty

Preoperative frailty will be measured by using the 9-item clinical frailty scale (CFS), translated to a Swedish version, the scale ranges from *1 – very vital* to *9 – terminally ill.* The Swedish version has been validated in persons > 65 years seeking care at an emergency department[24].

Delirium assessment

Delirium assessment will be performed using the observational five-item scale NU-DESC, where symptoms are rated from 0 to 2. A score of 2 or more indicates delirium [25]. A Swedish version of the Nu-DESC has been validated in persons undergoing cardiac surgery[26].

Postoperative recovery

Postoperative recovery will be measured with Swedish web version of Quality of Recovery, SwQoR. SwQoR is a psychometric evaluated including 24 different postoperative symptoms to be rated on 11-point numerical scales from 0, "never", to 10, "always"[27]. Global score ranging from 0 "excellent quality of postoperative recovery" to 240, "extremely poor quality of recovery", with cut-off values less than 31 at postoperative day (POD) 7 and less than 21 on POD 14 indicating good recovery [28]. Reliability and validity tests have presented evidence that it is suitable to use SwQoR in patients undergoing surgery [27].

Functional capacity

Functional status (Table 2) will be measured with the Swedish version of 12-item World Health Organization Disability Assessment Schedule 2.0 (WHODAS), ranges from 0−48, with increasing scores reflecting increasing disability[29].

For peer review only - http://bmjopen.bmj.com/site/about/guidelines.xhtml

Demographic and perioperative data

These include age, sex, educational level, MMSE score, comorbidities, American Society of Anaesthesiologists classification, pre-medication, choice of anesthetic drugs, duration of anesthesia and surgery, type of anaesthesia and surgery, intraoperative bleeding (mL), postoperative pain scores (numeric rating scale), intra- and postoperative opioids, early postoperative complications, and length of hospital stay.

Feasibility and patients' experiences of early postoperative cognitive recovery

Approximately 20-25 of the study participants will be included in an individual face-to-face interview exploring patients' experiences of early neurocognitive recovery and the use of Mindmore-P. The sample size will follow the concept of information power and will be determined within the process of data collection. Information power means that the more information the sample has, the lower the need to increase the amount of participants [30]. A semi-structured interview guide will be constructed and tested, examples on questions from the interview guide are "*Can you tell me about how you have been since you were discharged from the hospital?"* To gain a deeper understanding, probing questions will be asked, such as: "*Have you experienced any changes in your memory since the surgery?", "how has it been to plan and do different tasks after the surgery?"* and *"you mentioned …", "Could you tell me more about that?", "how do you mean* and *how would you describe that*? "and "*Can you give me any example?".* The participants will be asked to speak freely about their experiences of their postoperative neurocognitive recovery. All interviews will be audiotaped**.**

Procedure

Preoperative data collection

Eligible patients that consent to participate in the study will undergo the cognitive Mindmore-P test battery preoperatively. Before the test battery starts, we will inform about the Mindmore-P procedure and during the first test session a trained research nurse will be present in the room to assist with any technical help needed. Each test consists of an introduction both in audio and visualized on-screen, and the instructions can be repeated on demand. A practice trial is performed before the test battery starts. The digital cognitive test is expected to take less than 30 minutes. Depression, frailty, postoperative recovery and functional status will be measured after the cognitive testing is completed by the trained

For peer review only - http://bmjopen.bmj.com/site/about/guidelines.xhtml

research nurses (Table 2). It is estimated to take 15-20 minutes to complete the questionnaires. The day and time of day for preoperative data collection will be documented.

Postoperative data collection

We will assess delirium (Nu-DESC) twice a day (morning and afternoon) with start on POD 1 and until the patient is discharged from the hospital. Postoperative neurocognitive performance (Mindmore-P) and postoperative recovery (SwQoR) will be measured on postoperative day 1 or 2, and at the follow-up visit approximately 2-3 weeks after the surgery. We will measure depression (GDS-15), functional status (WHODAS) and usability of Mindmore-P (SUS) at the follow-up visit. Feasibility and experience of postoperative neurocognitive recovery will be assessed with interviews approximately one month after surgery in the patient's home or at the hospital (Table 2).

Table 2. Outcome measures

Outcome measures	Questionnaires	Preop	POD 1 or POD 2	Revisit at hospital 2-3 weeks postop	One month postop
**Dependent variable**<br>- **Postoperative neurocognitive recovery**	Mindmore-P	X	X	X	
**Independent variables**<br>- **Frailty**	Clinical Frailty Scale	X			
- **Depression**	GDS-15	X		X	
- **Functional capacity**	WHODAS-12	X		X	
- **Daily delirium assessment with start on day of surgery**	Nu-DESC				
- **Postoperative recovery**	SwQoR	X	X	X	
**Usability of Mindmore-P**	SUS			X	
**Feasibility: Experiences of neurocognitive recovery and digital assessment**	Qualitative interviews				X

Analysis

Descriptive statistics of demographic data will be presented with number, percentage, and mean (SD) or range, as appropriate. As recommended by Borchers et al review from 2021,

For peer review only - http://bmjopen.bmj.com/site/about/guidelines.xhtml

mild POCD (1-2 standard deviation [sd]) and major POCD (>2 sd) will be specified for each test result and POCD is present if at least two tests indicates decline[15].

Usability and attrition rates will be presented with descriptive statistics. Demographic characteristics, potential cofounders (i.e. patients characteristics, surgical and anaesthesiologic factors, preoperative depression (GDS), frailty (CFS), postoperative recovery (SwQoR), and functional status (WHODAS) will be compared between the groups i.e. delayed neurocognitive recovery or not, using parametric and non-parametric tests. To analyse intraindividual differences over time in cognitive performance, the same statistical tests will be used as well as multivariate logistic regression analysis. A p-value of <0.05 will indicate statistical significance in all analyses. We will analyse the interviews with thematic analysis[31].

Ethics and dissemination

All participants will obtain information about consent, confidentiality, voluntary basis of the study and the participant can refuse participation at any time. Each study participant will receive detailed information about duration and extent of the digital cognitive tests and the included outcomes, by the trained research nurses. The study participant can refuse to participate based on this information. Written consent will be obtained by the trained research nurses after they have received written and verbal information about the study. All study-related information will be stored securely digitally and physically at Ersta Hospital and at the university site. The local databases will be secured with password-protected access systems. The trial investigators will have access to the data, at present there is no plan to grant public access to participant-level dataset and statistical code after the study is finished. The project has been approved by the Swedish Ethical Review Authority (REC Reference: 2022-03593-01) and will follow the principles outlined in the 1964 Helsinki Declaration and its later amendments. Results from this study will be disseminated in peer-reviewed journals, scientific conferences and in social media. The trial investigators will follow the Uniform Requirements for Manuscripts[32]

For peer review only - http://bmjopen.bmj.com/site/about/guidelines.xhtml

Patient and public involvement

Patients or the public were not involved in the design, or conduct, or reporting, or dissemination plans of this research.

DISCUSSION

An evidence based, feasible and usable digital neurocognitive tool to is needed, especially as the growing population of older adults is increasing, and thereby the incidence of POCD is likely to rise. Both the American Geriatrics society[9] and The American Society of Anesthesiologists[1] recommend that older adults should be assessed for their cognitive performance prior to and after surgery. In line with this recommendation, we have conducted a comparability study between the analog ISPOCD test battery and the Mindmore-P digital test battery. The different test batteries were comparable, and the participants were overall positive to the digital cognitive test and thought the tests were useful and important to take if they would undergo surgery (Manuscript in progress). We will now take the next step evaluating Mindmore-P in a new context i.e., the clinical perioperative setting. This is an evaluation and adaptation process that follows the Medical Research Councils framework for designing and evaluating complex interventions [18]. Following a systematic process when measuring a complication that is not currently measured in clinical practice, i.e. postoperative neurocognitive performance, increases the transferability and quality of the evidence that will be produced. This pilot study is therefore of great importance to conduct.

Authors' contributions

AA contributed to planning of the study, study design, and the preparation of the manuscript and approved the final version. JE contributed to planning of the study, study design, and the preparation of the manuscript and approved the final version. AT, contributed to planning of the study, study design, and the preparation of the manuscript and approved the final version. LB contributed to planning of the study, study design, and the preparation of the manuscript and approved the final version. UN contributed to planning of the study, study design, and the preparation of the manuscript and approved the final version.

For peer review only - http://bmjopen.bmj.com/site/about/guidelines.xhtml

Funding statement

This work was supported by grants from the Strategic Research Area Health Care Science (grant number 2021-01095), The Erling-Persson Foundation (grant number 20190200) and Karolinska Institutet (grant number 2020-02641). These funding sources had no role in the design of the study and will have no role in its execution, analysis, data interpretation, or decision to submit results.

Postal address: Karolinska Institutet
Department of Neurobiology, Care sciences and Society /SFO-V
Division of Physiotherapy
Fack 23100
141 52 Huddinge
SWEDEN

Email: staffan.josephsson@ki.se

Competing interest statement.

None.

For peer review only

References

1. Mahanna-Gabrielli, E., et al., *State of the clinical science of perioperative brain health: report from the American Society of Anesthesiologists Brain Health Initiative Summit 2018.* Br J Anaesth, 2019. **123**(4): p. 464-478.
2. Nilsson, U., R. Gruen, and P.S. Myles, *Postoperative recovery: the importance of the team.* Anaesthesia, 2020. **75 Suppl 1**: p. e158-e164.
3. Nilsson, U., et al., *A journey to a new stable state-further development of the postoperative recovery concept from day surgical perspective: a qualitative study.* BMJ Open, 2020. **10**(9): p. e037755.
4. SPOR. 2022-10-20]; Swedish Perioperative Registry]. Available from: https://spor.se/spor/arsrapporter/.
5. Brown, C.L., et al., *Cognitive activity mediates the association between social activity and cognitive performance: A longitudinal study.* Psychol Aging, 2016. **31**(8): p. 831-846.
6. Zaninotto, P., et al., *Cognitive function trajectories and their determinants in older people: 8 years of follow-up in the English Longitudinal Study of Ageing.* J Epidemiol Community Health, 2018. **72**(8): p. 685-694.
7. Evered, L.A. and B.S. Silbert, *Postoperative cognitive dysfunction and noncardiac surgery.* Anesthesia & Analgesia, 2018. **127**(2): p. 496-505.
8. Vacas, S., et al., *Perioperative Brain Health in the Older Adult: A Patient Safety Imperative.* Anesth Analg, 2022. **135**(2): p. 316-328.
9. *American Geriatrics Society abstracted clinical practice guideline for postoperative delirium in older adults.* J Am Geriatr Soc, 2015. **63**(1): p. 142-50.
10. Ishizawa, Y., *Does Preoperative Cognitive Optimization Improve Postoperative Outcomes in the Elderly?* J Journal of Clinical Medicine, 2022. **11**(445).
11. Peden, C.J., et al., *Improving perioperative brain health: an expert consensus review of key actions for the perioperative care team.* British Journal of Anaesthesia, 2021. **126**(2): p. 423-432.
12. Krenk, L., L.S. Rasmussen, and H. Kehlet, *New insights into the pathophysiology of postoperative cognitive dysfunction.* Acta Anaesthesiologica Scandinavica, 2010. **54**(8): p. 951-956.
13. Patel, A., et al., *A Systematic Review and Meta-analysis Examining the Impact of Age on Perioperative Inflammatory Biomarkers.* Anesth Analg, 2022. **134**(4): p. 751-764.
14. Lin, X., et al., *The potential mechanism of postoperative cognitive dysfunction in older people.* Experimental gerontology, 2020. **130**: p. 110791.
15. Borchers, F., et al., *Methodology of measuring postoperative cognitive dysfunction: a systematic review.* British Journal of Anaesthesia, 2021. **126**: p. 1119e1127.
16. Amirpour, A., et al., *Is the analogue cognitive test from the ISPOCD equivalent to the digital cognitive test Mindmore? A protocol for a randomised cross-over study including qualitative interviews with self-reported healthy seniors.* BMJ Open, 2022. **12**(9): p. e062007.
17. Moller, J., et al., *Long-term postoperative cognitive dysfunction in the elderly: ISPOCD1 study.* The Lancet, 1998. **351**(9106): p. 857-861.
18. Skivington, K., et al., *A new framework for developing and evaluating complex interventions: update of Medical Research Council guidance.* Bmj, 2021. **374**: p. n2061.
19. Folstein, M.F., S.E. Folstein, and P.R. McHugh, *"Mini-mental state". A practical method for grading the cognitive state of patients for the clinician.* J Psychiatr Res, 1975. **12**(3): p. 189-98.
20. Lewis, J.R., *The system usability scale: past, present, and future.* International Journal of Human–Computer Interaction, 2018. **34**(7): p. 577-590.

For peer review only - http://bmjopen.bmj.com/site/about/guidelines.xhtml

21. Pocklington, C., et al., *The diagnostic accuracy of brief versions of the Geriatric Depression Scale: a systematic review and meta-analysis.* Int J Geriatr Psychiatry, 2016. **31**(8): p. 837-57.
22. Brown, L.M. and J.A. Schinka, *Development and initial validation of a 15-item informant version of the Geriatric Depression Scale.* Int J Geriatr Psychiatry, 2005. **20**(10): p. 911-8.
23. Conradsson, M., et al., *Usefulness of the Geriatric Depression Scale 15-item version among very old people with and without cognitive impairment.* Aging Ment Health, 2013. **17**(5): p. 638-45.
24. Hörlin, E., et al., *Inter-rater reliability of the Clinical Frailty Scale by staff members in a Swedish emergency department setting.* Acad Emerg Med, 2022.
25. Gaudreau, J.D., et al., *Fast, systematic, and continuous delirium assessment in hospitalized patients: the nursing delirium screening scale.* J Pain Symptom Manage, 2005. **29**(4): p. 368-75.
26. Lingehall, H.C., et al., *Validation of the Swedish version of the Nursing Delirium Screening Scale used in patients 70 years and older undergoing cardiac surgery.* J Clin Nurs, 2013. **22**(19-20): p. 2858-66.
27. Nilsson, U., K. Dahlberg, and M. Jaensson, *The Swedish Web Version of the Quality of Recovery Scale Adapted for Use in a Mobile App: Prospective Psychometric Evaluation Study.* JMIR mHealth uHealth, 2017. **5;12(e188)**.
28. Jaensson, M., et al., *Evaluation of postoperative recovery in day surgery patients using a mobile phone application: a multicentre randomized trial.* Br J Anaesth, 2017. **119**(5): p. 1030-1038.
29. Axelsson, E., et al., *The 12-item Self-Report World Health Organization Disability Assessment Schedule (WHODAS) 2.0 Administered Via the Internet to Individuals With Anxiety and Stress Disorders: A Psychometric Investigation Based on Data From Two Clinical Trials.* JMIR Ment Health, 2017. **4**(4): p. e58.
30. Malterud, K., V.D. Siersma, and A.D. Guassora, *Sample Size in Qualitative Interview Studies: Guided by Information Power.* Qual Health Res, 2016. **26**(13): p. 1753-1760.
31. Braun, V. and V. Clarke, *One size fits all? What counts as quality practice in (reflexive) thematic analysis?* Qualitative Research in Psychology, 2021. **18**(3): p. 328-352.
32. Barron, J.P., *The uniform requirements for manuscripts submitted to biomedical journals recommended by the International Committee of Medical Journal Editors.* Chest, 2006. **129**(4): p. 1098-1099.

For peer review only

For peer review only - http://bmjopen.bmj.com/site/about/guidelines.xhtml

SPIRIT 2013 Checklist: Recommended items to address in a clinical trial protocol and related documents*

Section/item	Item No	Description
**Administrative information**		
Title	1	Page 1, line 1-2.
Trial registration	2a	Page 1, line 13.
Protocol version	3	Page 1, line 15-18
Funding	4	Page 14, line 140-152
Roles and responsibilities	5a	Page 1 line 4-11
	5b, 5c	Page 13, line 329-335
**Introduction**		
Background and rationale	6a	Page 5-6.
Objectives	7	Page 6, line 134-151.
Trial design	8	Page 7, line 155
**Methods: Participants, interventions, and outcomes**		
Study setting	9	Page 7, Line 158
Eligibility criteria	10	Page 7, line 166-176
Outcomes	12	Page 7, line 180-182, Page 8-9, Page 10 line 229-248, Table 1
Participant timeline	13	Page 10, line 253-260, Page 11, line 261-272. Table 2
Sample size	14	Page 7, line 157-160
Recruitment	15	Page 7, line 157-165
**Methods: Data collection, management, and analysis**		

For peer review only - http://bmjopen.bmj.com/site/about/guidelines.xhtml

Data collection methods	18a	Page 10, line 253-260, Page 11, line 261-272. Table 2
	18b	Page 7 line 160-163
Data management	19	Page 12 line 294-301
Statistical methods	20a	Page 11 line 278-279
	20b	Page 12, line 290-291
	20c	Page 12 line 283
**Methods: Monitoring**		
Harms	22	Page 11
Auditing	23	Page 12-13
**Ethics and dissemination**		
Research ethics approval	24	Page 12, line 302-304
Consent or assent	26a	Page 7, line 160-163, Page 12 line 294-299
Confidentiality	27	Page 12 line 294-295
Declaration of interests	28	Page 14, line 342-356
Access to data	29	Page 12, line 301-303
Dissemination policy	31a	Page 12, line 306-307.
	31b	Page 12, line 307-308
	31c	Page 12, line 302-303

*It is strongly recommended that this checklist be read in conjunction with the SPIRIT 2013 Explanation & Elaboration for important clarification on the items. Amendments to the protocol should be tracked and dated. The SPIRIT checklist is copyrighted by the SPIRIT Group under the Creative Commons "Attribution-NonCommercial-NoDerivs 3.0 Unported" license.

For peer review only - http://bmjopen.bmj.com/site/about/guidelines.xhtml

BMJ Open

Usability and feasibility of a digital cognitive screening tool measuring older adults' early postoperative neurocognitive recovery: A protocol for a pilot study

Journal:	*BMJ Open*
Manuscript ID	bmjopen-2022-070404.R1
Article Type:	Protocol
Date Submitted by the Author:	27-Jan-2023
Complete List of Authors:	Amirpour, Anahita; Karolinska Institutet, Neurobiology, Care Sciences and Society<br>Eckerblad, Jeanette; Karolinska Institute,<br>Thorell , A; Karolinska institutet Department of Clinical Sciences Intervention and Technology,<br>Bergman, Lina; Karolinska Institute,<br>Nilsson, Ulrica; Karolinska Institutet, Department of Neurobiology, Care Sciences and Society, Karolinska Institutet Perioperative Medicine and Intensive Care; Karolinska Universitetssjukhuset, Perioperative Medicine and Intensive Care
**Primary Subject Heading**:	Anaesthesia
Secondary Subject Heading:	Nursing, Qualitative research, Surgery, Geriatric medicine
Keywords:	Adult anaesthesia < ANAESTHETICS, GERIATRIC MEDICINE, Adult surgery < SURGERY

SCHOLARONE™
Manuscripts

Usability and feasibility of a digital cognitive screening tool measuring older adults' early postoperative neurocognitive recovery: A protocol for a pilot study.

Anahita Amirpour[1], Jeanette Eckerblad [1], Anders Thorell [2], Lina Bergman [1], Ulrica Nilsson [1,3].

1. Department of Neurobiology, Care sciences and Society. Karolinska Institutet, Stockholm, Sweden.
2. Department of Clinical Science at Danderyds hospital, Karolinska Institutet, and Department of Surgery, Ersta Hospital, Stockholm, Sweden
3. Perioperative medicine and intensive care, Karolinska University Hospital, Stockholm, Sweden.

Trial registration number NCT05564195

Issue date 220801

Protocol amendment: 10

Authors: A.A, JE, AT, LB, UN

Revision chronology:

Corresponding Author

Anahita Amirpour

Division of Nursing

Department of Neurobiology, Care sciences and Society

Karolinska Institutet

Alfred Nobels allé, C4

14183 Huddinge, Sweden

Email: anahita.amirpour@ki.se

Phone: +46 8524 888 73

For peer review only

Key words: postoperative neurocognitive recovery, postoperative cognitive decline, geriatric medicine, adult anaesthesia.

Word count: 2979

For peer review only

For peer review only - http://bmjopen.bmj.com/site/about/guidelines.xhtml

ABSTRACT

Introduction Delayed neurocognitive recovery, also identified as early postoperative cognitive decline (POCD), is a common complication after surgery, with advanced age to be the most important risk factor. As the geriatric population is increasing worldwide, and number of older adults undergoing surgery continues to rise, so will the incidence of POCD. Only a small proportion using digital cognitive tests for measuring postoperative neurocognitive performance compared to analogue tests. This study aims to evaluate a digital cognitive screening tool, Mindmore-P, in a perioperative setting by determine its feasibility and usability and to compare preoperative cognition with early postoperative neurocognitive performance. Further, to determine associations between neurocognitive performance and perioperative factors as well as to explore patients' experiences of early neurocognitive recovery.

Methods and analysis: We will include fifty patients (age ≥60 years) undergoing elective abdominal surgery under general anesthesia. Cognitive functions will be measured with Mindmore-P preoperatively and on postoperative day (POD) 1 or 2 as well as 2-3 weeks after surgery. Preoperatively, frailty, (Clinical Frailty Scale), depression (Geriatric Depression Scale-15), functional status (WHODAS) and pre-recovery status (Swedish Quality of Recovery Scale, SwQoR) will be measured. Delirium will be assessed by Nu-DESC twice a day, with start on POD 1 and until the patient is discharged from the hospital. Outcomes at 2-3 weeks postoperatively are postoperative recovery (SwQoR), depression, functional status and usability (System Usability Scale) of Mindmore-P. Postoperative recovery will also be measured POD 1 or 2. We will also explore feasibility and experience of early postoperative neurocognitive recovery with interviews approximately one month after surgery.

Ethics and dissemination This study is approved by the Swedish Ethical Review Authority (REC Reference: 2022-03593-01) and will follow the principles outlined in the 1964 Helsinki Declaration and its later amendments. Results from this study will be disseminated in peer-reviewed journals, scientific conferences and in social media.

For peer review only - http://bmjopen.bmj.com/site/about/guidelines.xhtml

Strengths and limitations of this study:

- This study follows the Medical Research Councils framework for designing and evaluating complex interventions
- This study will provide evidence for the feasibility and usability of a digital neurocognitive tool to be used in a population of older persons undergoing surgery
- This pilot study will create a knowledge base for future interventions as previous studies are lacking
- A limitation is that the study only includes two follow ups

For peer review only

Introduction

The geriatric population is increasing worldwide[1]. Every fifth person in Sweden today is ≥65 years, and the number of persons of 80 years and over, is estimated to increase by 50% until 2050 [2, 3]. National statistics show that approximately 700 000 people undergo in-patient surgery, and almost 50% of those are performed on people ≥ 60 years[4]. However, hospital care after surgery remains short for the afflicted population; hence a major part of the postoperative recovery occurs after discharge from the hospital which [2] poses several risks for older people such as cognitive decline [1] .

Cognitive decline in older individuals has become an area of great concern, particularly given the impact of cognitive impairment on functional capacity[5]. Cognitive functions in late adulthood decline at different pace, and some cognitive domains are more prone to faster decline, such as executive functions, speed processing and memory [6]. Delayed neurocognitive recovery, earlier identified as postoperative cognitive decline (POCD), is an early postoperative cognitive complication, included in perioperative neurocognitive disorders. Delayed neurocognitive recovery is diagnosed up to 30 days after anaesthesia and surgery [1, 7] and is characterized by a gradual decreased performance in several cognitive domains such as memory, speed processing, executive function, and attention[1, 8]. The term POCD will be used in this article when referring to earlier research.

Preoperative risk factors for developing POCD after surgery are advanced age, pre-existing cognitive decline, visual and audial impairment, ongoing infection or serious illness [9]. Other risk factors are impaired functional status, lower level of educational attainment [7, 10, 11], alcohol abuse, electrolyte disturbances either medication-induced or due to dehydration, psychoactive medications, depression and frailty [10, 11]. Although no causality has been demonstrated, POCD is associated with poor postoperative outcomes including prolonged length of hospital stay, increased morbidity / mortality, and reduced quality of life, resulting in a significant burden on the individual, their family, as well as for the healthcare system. To date there is no existing pharmacological or non-pharmacological intervention to treat postoperative neurocognitive decline or decrease the associated symptoms [7, 11]. Moreover, results have been inconclusive regarding the role of intraoperative factors such as type of anesthesia, hypotension, cerebral saturation or choice of anesthetic drugs for the development of POCD [12]. One hypothetical mechanism is that older adults' have an unregulated acute inflammatory response to surgery[13]. So far, an explicit connection between POCD and

For peer review only - http://bmjopen.bmj.com/site/about/guidelines.xhtml

neuroinflammation due to the surgical trauma has been confirmed [7, 14].The American Geriatrics society's guidelines [9] recommend that every geriatric perioperative patient with risk factors, should be assessed for their cognitive performance prior surgery. Despite the potential benefits, it seems challenging to implement such a routine in regular clinical practice. There is therefore a need to develop and implement feasible measures to assess cognitive function in a busy clinical environment [11].

The methods used for measuring postoperative neurocognitive performance vary widely between studies, with only a small proportion, 13.5%, using digital cognitive tests or a combination of analog and digital tests [15]. To cover this gap we conducted a comparability study [16] between the digital cognitive test battery Mindmore Postoperative version, (Mindmore-P), and the analog International Study Group of Postoperative Cognitive Dysfunction (ISPOCD) test battery[17]. The study included 50 cognitively healthy participants aged 60 years and older. We found that the included tests in ISPOCD and Mindmore-P were comparable. Further, the participants were overall positive to digital testing and thought that neurocognitive testing was useful and important to assess if they would undergo surgery (Manuscript in progress). The next step, following Medical Research Councils framework for designing and evaluating complex interventions[18] is to assess predefined progression criteria to reduce uncertainty around recruitment, data collection, outcomes, sample size, and analysis in a future intervention study.

Aim

The aim of this study is to pilot test Mindmore-P, in a clinical perioperative setting. To achieve this, we intend:

I) To test the recruitment process and measure attrition rate.
II) To determine the usability and feasibility of Mindmore-P.
III) To explore the trajectory of delayed neurocognitive recovery by measuring the patient's preoperative and postoperative neurocognitive performance.
IV) To investigate if there are any associations between the dependent variable; delayed neurocognitive recovery and-the independent variables; delirium, preoperative frailty, symptoms of depression, postoperative delirium, physical function, and early postoperative recovery.
V) To determine patients' experiences of early neurocognitive recovery.

For peer review only - http://bmjopen.bmj.com/site/about/guidelines.xhtml

METHODS AND ANALYSIS

Study design

This is an observational feasibility study.

Participants, recruitment, and setting

We will include 50 patients undergoing elective abdominal surgery under general anesthesia at Ersta hospital in Stockholm, Sweden. Patient recruitment is planned to start in December 2022 and end in July 2023. The sample size is guided by a reported incidence of early cognitive decline at 1–2 weeks postoperatively of approximately 13-25% [17]. At the patient's preoperative outpatient visit, a research nurse will provide oral and written information about the study. The details of the study and its potential benefits and risks will be explained carefully to the patient. If the patients agree to participate in the study, they will undergo a Mini-mental state examination (MMSE) screening with the research nurse to examine if they are eligible for the study.

<u>Inclusion criteria:</u>

1) ≥60 years of age undergoing elective non-cardiac surgery with general anesthesia.
2) Duration of surgery ≥60 minutes
3) MMSE score ≥24.

<u>Exclusion criteria:</u>

1) MMSE score of ≤23 indicating cognitive impairment [19].
2) Inability to read and speak Swedish.
3) Uncorrected severe visual or auditory disorder, disease of the central nervous system, psychiatric diseases including alcohol abuse or drug dependence, current motor impairment in dominant hand, and colour vision deficiency.

Outcomes

Cognitive tests

The intervention is the digital cognitive test battery Mindmore-P (Table 1), which will assess neurocognitive performance. The test battery measures following cognitive domains; verbal episodic memory, executive functions, visuospatial function and attention.

For peer review only - http://bmjopen.bmj.com/site/about/guidelines.xhtml

Table 1. Included tests and cognitive domains in the Mindmore-P

**Cognitive Domain**	Mindmore-P
Verbal episodic memory	Consortium to Establish a Registry for Alzheimer´s Disease (CERAD)<br><br>A 10-word verbal learning test with 3 learning trials, a recall trial after ~7 min. Words are read and visually presented on the screen and the patient replies in speech.
Executive function, visuospatial function	Trail Making Test (TMT- A & B)<br><br>Each part consists of 25 circles with letters or numbers on the screen. In part A, the circles are numbered from 1 to 25. The patient is instructed to draw a line between them in ascending order of numbers, from 1 to 25, as rapidly as possible. Part B consists of 13 digits and 12 letters to be paired, and the test subject is instructed to draw a line between them in ascending number and letter order.
Executive function, selective attention	Stroop Colour-Word Test (SCW).<br><br>24 words spelling out the name of a colour which is printed in contrasting ink colours (e.g., the word “green” printed with red ink), and the participant is asked to tell the printed colour of the word rather than the actual meaning of the word.
Executive function, visuospatial function	Symbol Digits Processing Test (SDPT).<br><br>The test consists of nine symbol–digit pairings at the top of the screen, one symbol in the middle of the screen, and a 3 × 3 number grid at the bottom of the screen. The participant is required to associate the symbol in the middle to one of the digits on the grid below. As soon as a choice is made a new symbol appears. The final score is the number of correct matches in 90 seconds

The Mindmore-P is self-administered on a touch screen tablet (10.1″ 20-30 min Windows). and is a web-application running on a full-screen Chrome browser. The test is administered by using a capacitive touchscreen with features that record detailed information such as timing, pauses, and lifting of finger from the touchscreen. Speech recognition is used in the screening of verbal memory and language. Mindmore’s platform is CE certified and includes normative data adjusted for age, education and sex [20]

Usability

For peer review only

The usability (Table 2) of Mindmore-P will be assessed with the System Usability Scale (SUS). SUS consists of 10 items to be answered on a 5-point scale from “strongly agree” to “strongly disagree”. [21]

Depression screening

The Geriatric Depression Scale (GDS) is a widely used instrument to screen for depression in the older adult population[22]. The GDS-15 consists of 15 items with binary answer (yes/no) to questions such as “*Do you feel helpless*?” or “*Are you in good spirits most of the time*? [23]. The cut-off score is standardized with a score of 6 to the Swedish older adult population[24]. Every participant will answer the Swedish version of the GDS-15 before surgery and on postoperative day 14 at the revisit at the hospital.

Preoperative frailty

Preoperative frailty will be measured by using the 9-item clinical frailty scale (CFS), translated to a Swedish version, the scale ranges from *1 – very vital* to *9 – terminally ill.* The Swedish version has been validated in persons > 65 years seeking care at an emergency department[25].

Delirium assessment

Delirium assessment will be performed using the observational five-item scale NU-DESC, where symptoms are rated from 0 to 2. A score of 2 or more indicates delirium [26]. A Swedish version of the Nu-DESC has been validated in persons undergoing cardiac surgery[27].

Postoperative recovery

Postoperative recovery will be measured with Swedish web version of Quality of Recovery, SwQoR. SwQoR is a psychometric evaluated including 24 different symptoms such as fatigue, nausea, anxiety, headache, pain in the surgical wound, fever, to be rated on 11-point numerical scales from 0, “never”, to 10, “always”[28]. Global score ranging from 0 “excellent quality of postoperative recovery/ no symptoms” to 240, “extremely poor quality of recovery/severe symptoms ”, with cut-off values less than 31 at postoperative day (POD) 7 and less than 21 on POD 14 indicating good recovery [29]. Reliability and validity tests have presented evidence that it is suitable to use SwQoR in patients undergoing surgery [28].

Functional capacity

For peer review only - http://bmjopen.bmj.com/site/about/guidelines.xhtml

Functional status (Table 2) will be measured with the Swedish version of 12-item World Health Organization Disability Assessment Schedule 2.0 (WHODAS), ranges from 0−48, with increasing scores reflecting increasing disability[30].

Demographic and perioperative data

These include age, sex, educational level, MMSE score, comorbidities, American Society of Anaesthesiologists classification, pre-medication, choice of anesthetic drugs, duration of anesthesia and surgery, type of anaesthesia and surgery, intraoperative bleeding (mL), postoperative pain scores (numeric rating scale), intra- and postoperative opioids, early postoperative complications, and length of hospital stay.

Feasibility and patients' experiences of early postoperative cognitive recovery

Approximately 20-25 of the study participants will be included in an individual face-to-face interview exploring patients' experiences of early neurocognitive recovery and the use of Mindmore-P. The sample size will follow the concept of information power and will be determined within the process of data collection. Information power means that the more information the sample has, the lower the need to increase the amount of participants [31]. A semi-structured interview guide will be constructed and tested, examples on questions from the interview guide are "*Can you tell me about how you have been since you were discharged from the hospital?"* To gain a deeper understanding, probing questions will be asked, such as: "*Have you experienced any changes in your memory since the surgery?", "how has it been to plan and do different tasks after the surgery?"* and *"you mentioned …", "Could you tell me more about that?", "how do you mean* and *how would you describe that*? "and "*Can you give me any example?".* The participants will be asked to speak freely about their experiences of their postoperative neurocognitive recovery. All interviews will be audiotaped**.**

Procedure

Preoperative data collection

Eligible patients that consent to participate in the study will undergo the cognitive Mindmore-P test battery preoperatively. Before the test battery starts, we will inform about the Mindmore-P procedure and during the first test session a trained research nurse will be present in the room to assist with any technical help needed. Each test consists of an introduction both in audio and visualized on-screen, and the instructions can be repeated on

For peer review only

For peer review only - http://bmjopen.bmj.com/site/about/guidelines.xhtml

demand. A practice trial is performed before the test battery starts. The digital cognitive test is expected to take less than 30 minutes. Depression, frailty, postoperative recovery (except for three questions about the surgical wound) and functional status will be measured after the cognitive testing is completed by the trained research nurses (Table 2). It is estimated to take 15-20 minutes to complete the questionnaires. The day and time of day for preoperative data collection will be documented.

Postoperative data collection

We will assess delirium (Nu-DESC) twice a day (morning and afternoon) with start on POD 1 and until the patient is discharged from the hospital. Postoperative neurocognitive performance (Mindmore-P) and postoperative recovery (SwQoR) will be measured on postoperative day 1 or 2, and at the follow-up visit approximately 2 weeks after the surgery. We will measure depression (GDS-15), functional status (WHODAS) and usability of Mindmore-P (SUS) at the follow-up visit. Feasibility and experience of postoperative neurocognitive recovery will be assessed with interviews approximately one month after surgery in the patient's home or at the hospital (Table 2).

Table 2. Outcome measures

Outcome measures	Questionnaires	Preop	POD 1 or POD 2	Revisit at hospital POD 14 + 0-5 days	One month postop
**Dependent variable**<br>- **Postoperative neurocognitive recovery**	Mindmore-P	X	X	X	
**Independent variables**<br>- **Frailty**	Clinical Frailty Scale	X			
- **Depression**	GDS-15	X		X	
- **Functional capacity**	WHODAS-12	X		X	
- **Daily delirium assessment with start on day of surgery**	Nu-DESC				
- **Postoperative recovery**	SwQoR	X	X	X	
**Usability of Mindmore-P**	SUS			X	
**Feasibility: Experiences of neurocognitive recovery and digital assessment**	Qualitative interviews				X

Analysis

For peer review only - http://bmjopen.bmj.com/site/about/guidelines.xhtml

Descriptive statistics of demographic data will be presented with number, percentage, and mean (SD) or range, as appropriate. A within-subject analysis a standardized reliable change Z-score for each postoperative neurocognitive test (n=4) will be calculated, based on the difference between patient's test results at baseline at baseline (i.e. preoperative) vs. postoperative tests results, and normalize using Mindmore's normative data for each specific test [20] As recommended by Borchers et al review from 2021, mild POCD (1-2 standard deviation [sd]) and major POCD (>2 sd) will be specified for each test result and POCD is present if at least two tests indicates decline [15].

Usability and attrition rates will be presented with descriptive statistics. Demographic characteristics, potential cofounders (i.e. patients characteristics, surgical and anaesthesiologic factors, preoperative depression (GDS), frailty (CFS), postoperative recovery (SwQoR), and functional status (WHODAS) will be compared between the groups i.e. delayed neurocognitive recovery or not, using parametric and non-parametric tests. To analyse intraindividual differences over time in cognitive performance, the same statistical tests will be used as well as multivariate logistic regression analysis. A p-value of <0.05 will indicate statistical significance in all analyses. We will analyse the interviews with thematic analysis[32].

Ethics and dissemination

All participants will obtain information about consent, confidentiality, voluntary basis of the study and the participant can refuse participation at any time. Each study participant will receive detailed information about duration and extent of the digital cognitive tests and the included outcomes, by the trained research nurses. The study participant can refuse to participate based on this information. Written consent will be obtained by the trained research nurses after they have received written and verbal information about the study. All study-related information will be stored securely digitally and physically at Ersta Hospital and at the university site. The local databases will be secured with password-protected access systems. The trial investigators will have access to the data, at present there is no plan to grant public access to participant-level dataset and statistical code after the study is finished. The project has been approved by the Swedish Ethical Review Authority (REC Reference: 2022-03593-01) and will follow the principles outlined in the 1964 Helsinki Declaration and its later amendments. Results from this study will be disseminated in peer-reviewed journals,

For peer review only - http://bmjopen.bmj.com/site/about/guidelines.xhtml

scientific conferences and in social media. The trial investigators will follow the Uniform Requirements for Manuscripts[33]

Patient and public involvement

Patients or the public were not involved in the design, or conduct, or reporting, or dissemination plans of this research.

DISCUSSION

An evidence based, feasible and usable digital neurocognitive tool to is needed, especially as the growing population of older adults is increasing, and thereby the incidence of POCD is likely to rise. Both the American Geriatrics society[9] and The American Society of Anesthesiologists[1] recommend that older adults should be assessed for their cognitive performance prior to and after surgery. In line with this recommendation, we have conducted a comparability study between the analog ISPOCD test battery and the Mindmore-P digital test battery. The different test batteries were comparable, and the participants were overall positive to the digital cognitive test and thought the tests were useful and important to take if they would undergo surgery (Manuscript in progress). We will now take the next step evaluating Mindmore-P in a new context i.e., the clinical perioperative setting. This is an evaluation and adaptation process that follows the Medical Research Councils framework for designing and evaluating complex interventions [18]. Following a systematic process when measuring a complication that is not currently measured in clinical practice, i.e. postoperative neurocognitive performance, increases the transferability and quality of the evidence that will be produced. This pilot study is therefore of great importance to conduct as it will determine not only the feasibility and usefulness of Mindmore-P, but it will also guide us in the choice of independent variables, the primary outcome and the sample size in a future study.

Authors' contributions

AA contributed to planning of the study, study design, and the preparation of the manuscript and approved the final version. JE contributed to planning of the study, study design, and the

For peer review only - http://bmjopen.bmj.com/site/about/guidelines.xhtml

preparation of the manuscript and approved the final version. AT, contributed to planning of the study, study design, and the preparation of the manuscript and approved the final version. LB contributed to planning of the study, study design, and the preparation of the manuscript and approved the final version. UN contributed to planning of the study, study design, and the preparation of the manuscript and approved the final version.

Funding statement

This work was supported by grants from the Strategic Research Area Health Care Science (grant number 2021-01095), The Erling-Persson Foundation (grant number 20190200) and Karolinska Institutet (grant number 2020-02641). These funding sources had no role in the design of the study and will have no role in its execution, analysis, data interpretation, or decision to submit results.

Postal address: Karolinska Institutet
Department of Neurobiology, Care sciences and Society /SFO-V
Division of Physiotherapy
Fack 23100
141 52 Huddinge
SWEDEN

Email: staffan.josephsson@ki.se

For peer review only

Competing interest statement.

None.

For peer review only - http://bmjopen.bmj.com/site/about/guidelines.xhtml

References

1. Mahanna-Gabrielli, E., et al., *State of the clinical science of perioperative brain health: report from the American Society of Anesthesiologists Brain Health Initiative Summit 2018.* Br J Anaesth, 2019. **123**(4): p. 464-478.
2. Nilsson, U., R. Gruen, and P.S. Myles, *Postoperative recovery: the importance of the team.* Anaesthesia, 2020. **75 Suppl 1**: p. e158-e164.
3. Nilsson, U., et al., *A journey to a new stable state-further development of the postoperative recovery concept from day surgical perspective: a qualitative study.* BMJ Open, 2020. **10**(9): p. e037755.
4. SPOR. 2022-10-20]; Swedish Perioperative Registry]. Available from: https://spor.se/spor/arsrapporter/.
5. Brown, C.L., et al., *Cognitive activity mediates the association between social activity and cognitive performance: A longitudinal study.* Psychol Aging, 2016. **31**(8): p. 831-846.
6. Zaninotto, P., et al., *Cognitive function trajectories and their determinants in older people: 8 years of follow-up in the English Longitudinal Study of Ageing.* J Epidemiol Community Health, 2018. **72**(8): p. 685-694.
7. Evered, L.A. and B.S. Silbert, *Postoperative cognitive dysfunction and noncardiac surgery.* Anesthesia & Analgesia, 2018. **127**(2): p. 496-505.
8. Vacas, S., et al., *Perioperative Brain Health in the Older Adult: A Patient Safety Imperative.* Anesth Analg, 2022. **135**(2): p. 316-328.
9. *American Geriatrics Society abstracted clinical practice guideline for postoperative delirium in older adults.* J Am Geriatr Soc, 2015. **63**(1): p. 142-50.
10. Ishizawa, Y., *Does Preoperative Cognitive Optimization Improve Postoperative Outcomes in the Elderly?* J Journal of Clinical Medicine, 2022. **11**(445).
11. Peden, C.J., et al., *Improving perioperative brain health: an expert consensus review of key actions for the perioperative care team.* British Journal of Anaesthesia, 2021. **126**(2): p. 423-432.
12. Krenk, L., L.S. Rasmussen, and H. Kehlet, *New insights into the pathophysiology of postoperative cognitive dysfunction.* Acta Anaesthesiologica Scandinavica, 2010. **54**(8): p. 951-956.
13. Patel, A., et al., *A Systematic Review and Meta-analysis Examining the Impact of Age on Perioperative Inflammatory Biomarkers.* Anesth Analg, 2022. **134**(4): p. 751-764.
14. Lin, X., et al., *The potential mechanism of postoperative cognitive dysfunction in older people.* Experimental gerontology, 2020. **130**: p. 110791.
15. Borchers, F., et al., *Methodology of measuring postoperative cognitive dysfunction: a systematic review.* British Journal of Anaesthesia, 2021. **126**: p. 1119e1127.
16. Amirpour, A., et al., *Is the analogue cognitive test from the ISPOCD equivalent to the digital cognitive test Mindmore? A protocol for a randomised cross-over study including qualitative interviews with self-reported healthy seniors.* BMJ Open, 2022. **12**(9): p. e062007.
17. Moller, J., et al., *Long-term postoperative cognitive dysfunction in the elderly: ISPOCD1 study.* The Lancet, 1998. **351**(9106): p. 857-861.
18. Skivington, K., et al., *A new framework for developing and evaluating complex interventions: update of Medical Research Council guidance.* Bmj, 2021. **374**: p. n2061.
19. Folstein, M.F., S.E. Folstein, and P.R. McHugh, *"Mini-mental state". A practical method for grading the cognitive state of patients for the clinician.* J Psychiatr Res, 1975. **12**(3): p. 189-98.
20. van den Hurk, W., et al., *Swedish Normative Data for Mindmore: A Comprehensive Cognitive Screening Battery, Both Digital and Self-Administrated.* Journal of the International Neuropsychological Society, 2022. **28**(2): p. 188-202.

For peer review only

For peer review only - http://bmjopen.bmj.com/site/about/guidelines.xhtml

21. Lewis, J.R., *The system usability scale: past, present, and future.* International Journal of Human–Computer Interaction, 2018. **34**(7): p. 577-590.
22. Pocklington, C., et al., *The diagnostic accuracy of brief versions of the Geriatric Depression Scale: a systematic review and meta-analysis.* Int J Geriatr Psychiatry, 2016. **31**(8): p. 837-57.
23. Brown, L.M. and J.A. Schinka, *Development and initial validation of a 15-item informant version of the Geriatric Depression Scale.* Int J Geriatr Psychiatry, 2005. **20**(10): p. 911-8.
24. Conradsson, M., et al., *Usefulness of the Geriatric Depression Scale 15-item version among very old people with and without cognitive impairment.* Aging Ment Health, 2013. **17**(5): p. 638-45.
25. Hörlin, E., et al., *Inter-rater reliability of the Clinical Frailty Scale by staff members in a Swedish emergency department setting.* Acad Emerg Med, 2022.
26. Gaudreau, J.D., et al., *Fast, systematic, and continuous delirium assessment in hospitalized patients: the nursing delirium screening scale.* J Pain Symptom Manage, 2005. **29**(4): p. 368-75.
27. Lingehall, H.C., et al., *Validation of the Swedish version of the Nursing Delirium Screening Scale used in patients 70 years and older undergoing cardiac surgery.* J Clin Nurs, 2013. **22**(19-20): p. 2858-66.
28. Nilsson, U., K. Dahlberg, and M. Jaensson, *The Swedish Web Version of the Quality of Recovery Scale Adapted for Use in a Mobile App: Prospective Psychometric Evaluation Study.* JMIR mHealth uHealth, 2017. **5;12(e188)**.
29. Jaensson, M., et al., *Evaluation of postoperative recovery in day surgery patients using a mobile phone application: a multicentre randomized trial.* Br J Anaesth, 2017. **119**(5): p. 1030-1038.
30. Axelsson, E., et al., *The 12-item Self-Report World Health Organization Disability Assessment Schedule (WHODAS) 2.0 Administered Via the Internet to Individuals With Anxiety and Stress Disorders: A Psychometric Investigation Based on Data From Two Clinical Trials.* JMIR Ment Health, 2017. **4**(4): p. e58.
31. Malterud, K., V.D. Siersma, and A.D. Guassora, *Sample Size in Qualitative Interview Studies: Guided by Information Power.* Qual Health Res, 2016. **26**(13): p. 1753-1760.
32. Braun, V. and V. Clarke, *One size fits all? What counts as quality practice in (reflexive) thematic analysis?* Qualitative Research in Psychology, 2021. **18**(3): p. 328-352.
33. Barron, J.P., *The uniform requirements for manuscripts submitted to biomedical journals recommended by the International Committee of Medical Journal Editors.* Chest, 2006. **129**(4): p. 1098-1099.

For peer review only

STANDARD PROTOCOL ITEMS: RECOMMENDATIONS FOR INTERVENTIONAL TRIALS

SPIRIT 2013 Checklist: Recommended items to address in a clinical trial protocol and related documents*

Section/item	Item No	Description
**Administrative information**		
Title	1	Page 1, line 1-2.
Trial registration	2a	Page 1, line 13.
Protocol version	3	Page 1, line 15-18
Funding	4	Page 14, line 140-152
Roles and responsibilities	5a	Page 1 line 4-11
	5b, 5c	Page 13, line 329-335
**Introduction**		
Background and rationale	6a	Page 5-6.
Objectives	7	Page 6, line 134-151.
Trial design	8	Page 7, line 155
**Methods: Participants, interventions, and outcomes**		
Study setting	9	Page 7, Line 158
Eligibility criteria	10	Page 7, line 166-176
Outcomes	12	Page 7, line 180-182, Page 8-9, Page 10 line 229-248, Table 1
Participant timeline	13	Page 10, line 253-260, Page 11, line 261-272. Table 2
Sample size	14	Page 7, line 157-160
Recruitment	15	Page 7, line 157-165
**Methods: Data collection, management, and analysis**		

For peer review only

Data collection methods	18a	Page 10, line 253-260, Page 11, line 261-272. Table 2
	18b	Page 7 line 160-163
Data management	19	Page 12 line 294-301
Statistical methods	20a	Page 11 line 278-279
	20b	Page 12, line 290-291
	20c	Page 12 line 283
**Methods: Monitoring**		
Harms	22	Page 11
Auditing	23	Page 12-13
**Ethics and dissemination**		
Research ethics approval	24	Page 12, line 302-304
Consent or assent	26a	Page 7, line 160-163, Page 12 line 294-299
Confidentiality	27	Page 12 line 294-295
Declaration of interests	28	Page 14, line 342-356
Access to data	29	Page 12, line 301-303
Dissemination policy	31a	Page 12, line 306-307.
	31b	Page 12, line 307-308
	31c	Page 12, line 302-303

*It is strongly recommended that this checklist be read in conjunction with the SPIRIT 2013 Explanation & Elaboration for important clarification on the items. Amendments to the protocol should be tracked and dated. The SPIRIT checklist is copyrighted by the SPIRIT Group under the Creative Commons "Attribution-NonCommercial-NoDerivs 3.0 Unported" license.

For peer review only - http://bmjopen.bmj.com/site/about/guidelines.xhtml
